# Association of amyloid-beta with depression or depressive symptoms in older adults without dementia: a systematic review and meta-analysis

**DOI:** 10.1038/s41398-024-02739-9

**Published:** 2024-01-15

**Authors:** Emma L. Twait, Jen-Hao Wu, Maria Kamarioti, Maartje Basten, Wiesje M. van der Flier, Lotte Gerritsen, Mirjam I. Geerlings

**Affiliations:** 1 Amsterdam UMC, location Vrije Universiteit, Department of General Practice, Van der Boechorststraat 7, Amsterdam, The Netherlands; 2 Amsterdam Public Health; Aging & Later life, and Personalized Medicine, Amsterdam, The Netherlands; 3 Amsterdam Neuroscience; Neurodegeneration, and Mood, Anxiety, Psychosis, Stress, and Sleep, Amsterdam, The Netherlands; 4 Julius Center for Health Sciences and Primary Care, University Medical Center Utrecht and Utrecht University, Utrecht, The Netherlands; 5 Alzheimer Center Amsterdam, Neurology, Epidemiology and Data Science, Vrije Universiteit Amsterdam, Amsterdam UMC location VUmc, Amsterdam, The Netherlands; 6 Amsterdam Neuroscience, Neurodegeneration, Amsterdam, The Netherlands; 7 Epidemiology and Data Science, Vrije Universiteit Amsterdam, Amsterdam UMC location VUmc, Amsterdam, The Netherlands; 8 Department of Psychology, Utrecht University, Utrecht, The Netherlands; 9 Amsterdam UMC, location University of Amsterdam, Department of General Practice, Meibergdreef 9, Amsterdam, The Netherlands

**Keywords:** Depression, Prognostic markers

## Abstract

Several lines of evidence have indicated that depression might be a prodromal symptom of Alzheimer’s disease (AD). This systematic review and meta-analysis investigated the cross-sectional association between amyloid-beta, one of the key pathologies defining AD, and depression or depressive symptoms in older adults without dementia. A systematic search in PubMed yielded 689 peer-reviewed articles. After full-text screening, nine CSF studies, 11 PET studies, and five plasma studies were included. No association between amyloid-beta and depression or depressive symptoms were found using cerebrospinal fluid (CSF) (0.15; 95% CI: −0.08; 0.37), positron emission topography (PET) (Cohen’s *d:* 0.09; 95% CI: −0.05; 0.24), or plasma (−0.01; 95% CI: −0.23; 0.22). However, subgroup analyses revealed an association in plasma studies of individuals with cognitive impairment. A trend of an association was found in the studies using CSF and PET. This systematic review and meta-analysis suggested that depressive symptoms may be part of the prodromal stage of dementia.

## Introduction

Depression is one of the leading mental disorders seen in older individuals, which can lead to decreased quality of life, disability, and higher comorbidity from other medical conditions [1, 2]. A recent meta-analysis found a pooled prevalence of 32% of depression in later life [3]. Further, late-life depression is associated with an increased risk of all-cause dementia and Alzheimer’s disease (AD) [4–7]. Although the pathways are not fully understood, this increased risk could be explained by late-life depression being a risk factor for the development of AD [6, 8]; however, recent studies suggest that late-life depression may be part of the prodromal stage for AD [9–13].

AD pathophysiology develops years before cognitive decline begins (i.e., the preclinical stage) and may drive late-life depressive symptoms [14]. The main pathological hallmark of AD is amyloid-β (Aβ) peptide aggregation which forms amyloid plaques [15, 16]. In clinical practice, Aβ positron-emission tomography (PET) scans and measurement of Aβ in CSF are validated methods for identifying AD pathophysiology [17, 18]. Plasma Aβ level has also demonstrated potential clinical importance in detecting brain Aβ burden, and recent blood assays have been developed that are more sensitive to quantify Aβ [18]. Alongside being attributed to AD, plasma and CSF Aβ levels have also been suggested to be altered in individuals with depression in several studies, suggesting that both syndromes may share underlying pathophysiology.

A previous systematic review and meta-analysis by Nascimento et al. [19] on 12 studies reported significantly higher plasma Aβ40/Aβ42 ratio (i.e., higher Aβ burden) in plasma in those with depression, but no significant differences in CSF Aβ42 were found. However, some studies published after the review had contradictory results. One longitudinal study comparing individuals diagnosed with depression and healthy controls found significantly lower CSF Aβ42 levels at baseline in the individuals with depression [20]. Additionally, recently developed ELISA plasma assays are more sensitive, warranting for an updated meta-analysis assessing plasma. The previous systematic review and meta-analysis also did not include PET studies. Lastly, the previous review did not assess cognitive status, which may determine one’s stage in preclinical or prodromal AD, as several studies included individuals with both mild cognitive impairment (MCI) and normal cognition.

As the relationship between Aβ and depression may differ based on Aβ assessment and cognitive status (i.e., being in either preclinical or prodromal AD), an updated meta-analysis is warranted, including PET studies, more recent studies assessing plasma Aβ with more sensitive techniques, as well as examining possible differences based on cognitive status. In this systematic review and meta-analysis, we aimed to examine the cross-sectional association of Aβ burden (measured by CSF, PET, or plasma) with depression or depressive symptoms in older adults without dementia to assess possible biological mechanisms of late-life depression.

## Methods

This systematic review and meta-analysis was conducted and reported following the PRISMA guidelines [21]. The review was not registered on PROSPERO as data collection had already been performed.

### Search and study selection

A search string including the terms depression, amyloid, method of amyloid measurement (i.e., CSF, PET, or plasma), and their synonyms (Supplementary Info 1) was developed for PubMed, focusing on older adults without dementia. The original search was performed on May 14, 2021, and duplicate results from our search were removed with EndNote (v. 20.2) (The EndNote Team, 2013) reference management software. Subsequently, two reviewers (E.T. and M.K.) independently screened titles and abstracts using the Rayyan app [22] to assess eligibility, blinded by each other’s decisions. On May 18, 2022, and then subsequently on July 3, 2023, an updated search was performed by two reviewers (E.T. and J.W.) using the same screening strategies listed above. Full texts of the remaining articles were retrieved and screened against eligibility criteria. Any disagreements were resolved by discussion between the two reviewers (E.T. and J.W.). Snowballing and reverse snowballing were performed by scanning the reference lists of the included articles for any other publications of interest as well as searching Scopus for other works that cited the included articles.

### Eligibility criteria

Studies reporting an association between Aβ burden (measured by either CSF, PET, or plasma) and depression diagnosis (determined by a clinical depression diagnosis from medical history or based on established depression evaluation criteria) or depressive symptoms (assessed with a depressive symptom questionnaire) were eligible for inclusion. Eligible studies i) presented observational cross-sectional associations or ii) were longitudinal in design but reported baseline characteristics and associations. Only articles reporting associations in non-demented older adults (i.e., mean age of study population ≥50 years old) were included. There were no criteria for the language or publication date of the study. In addition, studies with insufficient information for calculating an effect size were excluded if the corresponding author could not provide the information needed. If multiple articles used the same cohort to investigate the association, the study containing the largest number of study participants was included.

### Data extraction and risk of bias assessment

Information extracted from the selected articles was the cohort, size of the study sample, baseline characteristics, Aβ measurement (CSF, PET, or plasma), Aβ burden classification (continuous or categorical), depression assessment criteria (clinical diagnosis or depressive symptoms), covariate adjustment (whether the study controlled for age, sex, education, or other factors), and the effect size between Aβ and depression or depressive symptoms.

The risk of bias was assessed using an adjusted version of the Newcastle-Ottawa Quality Assessment Scale for Cohort Studies (Supplementary Info 2), in which the included studies were rated with stars based on nine criteria divided into three sections: the quality of the study population selection, the comparability of cohorts based on the study design or analysis, and the quality of outcome assessment.

### Data extraction and risk of bias assessment

Statistical analyses were performed using R version 4.0.5 (RStudio, 2022). Based on means and standard deviations, odds ratios, t-tests, chi-squares, and beta coefficients, these metrics from each study were transformed into standardized mean differences (i.e., Cohen’s d) using the *esc* package in R [23]. Notably, lower CSF or plasma amyloid levels indicate a higher brain amyloid burden [24, 25]; therefore, effect sizes were reversed if studies measured Aβ via CSF or plasma. By reversing the effect size in such cases, a positive Cohen’s d would represent an association between higher Aβ burden and depression or depressive symptoms. Considering the possible heterogeneity between studies, such as depression assessment criteria, it might not be reasonable to assume a common effect across included studies. Therefore, the pooled estimate was calculated using a random-effects model [26] using the *meta* and *metafor* packages [27, 28].

Several studies reported multiple Aβ metrics from the same subjects (e.g., reporting both Aβ40, Aβ42, and their ratio, continuous and categorical scales of Aβ burden, depression assessed based on clinical diagnosis and depressive symptoms, and both adjusted and unadjusted associations). To prevent including one study multiple times in the meta-analysis, a prioritization was made to include only one effect size from each study. We chose a continuous scale of Aβ, depression assessment based on clinical diagnosis, Aβ42/40 ratio, and analyses adjusted for covariates as our prioritization criteria for the meta-analysis, to produce a more clinically relevant result and reduce possible heterogeneity. Therefore, no studies were included twice.

Cochran’s Q test and I^2^ statistics were used to test heterogeneity. Based on the Cochrane Handbook [29], 30–60%, 50–90%, and more than 75% were rated, respectively, as moderate, substantial, or considerable heterogeneity. To assess the risk of publication bias, visual inspection of funnel plots and Egger’s t-test were performed. Subgroup analyses were done to explore biological and methodological heterogeneity. Subgroups were stratified according to: adjusted/unadjusted for covariates, continuous vs. categorical assessment of Aβ burden, depression assessment (based on clinical diagnosis/depressive symptoms), cohort origin (general population/clinical settings), and if participants were cognitively impaired or not. Meta-regression was performed to assess if sex/gender distribution, prevalence of APOE e4 allele genotype, or prevalence of cognitively impaired individuals affected the results. For all tests, a *p*-value < 0.05 was considered statistically significant.

## Results

**Fig. 1:**
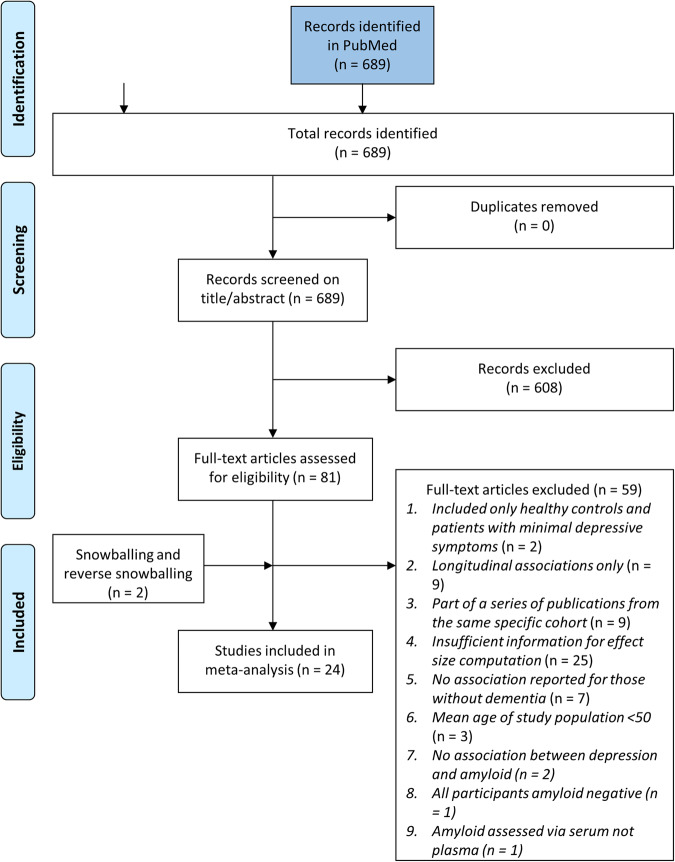
PRISMA flow chart. Flow chart of the original literature search.

Following the removal of duplicates, 689 articles were retrieved, of which 81 articles were assessed full-text for eligibility (Fig. 1). After the full-text screening, our meta-analysis included 24 studies [30–53] (Fig. 1). There were nine CSF studies, 11 PET studies, and five plasma studies. One study [42] reported both PET and plasma metrics. However, as the main analysis was stratified by amyloid assessment method, this study was not included twice in one analysis.

The demographics of the participants from each study are presented in Table 1. There was a total of 2706 study participants from the nine included CSF studies, 6418 participants from the 11 PET studies, and 2312 participants from the five plasma studies. For all 24 included studies, sample size varied from 28 to 4492, mean age ranged from 61 to 78 years, percentage of female participants ranged from 26 to 100%, mean education ranged from three to 17 years, prevalence of an APOE e4 allele ranged from 14 to 39%, if reported. Prevalence of a clinical diagnosis or high depressive symptomology ranged from three to 71% in the studies. All of the studies used depression as the outcome. The origin of the study cohort varied from general population to clinical settings, such as hospitals or memory clinics. Six (67%) of the nine CSF studies used a clinical diagnosis of depression, whereas seven (64%) of the 11 PET studies and two of the five (40%) of the plasma studies used a clinical diagnosis of depression (Table 2). One (11%) of the nine CSF studies, two (18%) of the 11 PET studies, and none (0%) of the plasma studies assessed amyloid categorically. Four (44%) of the nine CSF studies, four (36%) of the 11 PET studies, and four (80%) of the five plasma studies controlled for one or more covariates, such as age, sex/gender, and education or used age-matched controls. Of the 11 PET studies, seven studies (64%) used a ^18^F tracer and four (36%) studies used a ^11^C tracer. Of the nine CSF studies, six (67%) studies reported only Aβ42, two (22%) studies reported both Aβ42 and Aβ40, and one (11%) study reported the Aβ42/40 ratio. Of the five plasma studies, two (40%) studies reported both Aβ42 and Aβ40, two studies (40%) reported Aβ42, Aβ40, and the ratio, and one study (20%) reported only Aβ42.

**Table 1:** Characteristics of the participants of the included studies in the meta-analysis.

Study (Year, Country)	Cohort origin	Sample size	Age (Mean ± SD in years)	Sex/gender (% women)	Education (Mean ± SD in years)	APOE e4 allele presence (%)	Prevalence of depression diagnosis or high depressive symptoms (%)	Cognitive impairment (%)
CSF studies								
Diniz et al. [43] (2014, Brazil)	Hospital	50	70 ± 5	66%	12 ± 6	–	50%	20% MCI
Gudmundsson et al. [45] (2007, Sweden)	Population	84	73 ± 3	100%	–	–	17%	None
Hertze et al. [46] (2010, Sweden)	Memory clinic	66	69 ± 13	62%	–	27%	42%	None
Hu et al. [51] (2022, China)	Hospital	1005	61 ± 10	40%	10 ± 4	15%	12% HAMD ≥ 7	39% SCD
Krell-Roesch et al. [31] (2022, USA)	Population	784	73 ± 7	43%	14 ± 3	27%	9% BDI ≥ 13	11% MCI
Marquié et al. [52] (2023, Spain)	Memory clinic	500	73 ± 8	55%	8 ± 5	26%	50% prevalence on NPI-Q	83% MCI
Pomara et al. [35] (2012, USA)	Population	47	67 ± 6	47%	17 ± 3	34%	60%	None
Reis et al. [36] (2012, Brazil)	Population	28	71 ± 6	89%	5 ± 4	–	71%	75% MDD patients SCD
Siafarikas et al. [37] (2021, Norway)	Hospital	142	68 ± 8	51%	13 ± 3	–	25%	46% with MCI/SCD
PET studies								
Babulal et al. [41] (2020, USA)	Research center	301	70 ± 8	57%	16 ± 2	33%	13%	None
Byun et al. [42] (2016, South Korea)	Hospital	56	70 ± 6	61%	10 ± 5	14%	52%	48% MCI in MDD group
Donovan et al. [47] (2015, USA)	Population	248	74 ± 6	60%	–	–	6%	None
Feng et al. [48] (2023, Canada)	Hospital	133	78 ± 7	57%	–	31%	38%	100% MCI
Kumar et al. [32] (2011, USA)	Population	39	67 ± 7	56%	16 ± 3	–	51%	5% MCI in MDD group
Lewis et al. [33] (2022, USA)	Hospital	4492	71 ± 5	59%	–	–	3% GDS > 5	None
Moriguchi et al. [34] (2021, Japan)	Hospital	40	72 ± 7	75%	13 ± 2	–	50%	None
Touron et al. [49] (2022, France)	Population	135	69 ± 4	61%	13 ± 3	27%	57% GDS > 0	None
Wang et al. [39] (2021, South Korea)	Hospital	235	70 ± 9	71%	13 ± 4	23%	50%	None
Weigand et al. [50] (2022, USA)	Population	703	73 ± 8	49%	16 ± 2	39%	–	31% MCI
Wu et al. [40] (2014, Taiwan)	Population	36	69 ± 6	81%	8 ± 4	19%	69%	None
Plasma studies								
Byun et al. [42] (2016, South Korea)	Hospital	56	70 ± 6	61%	10 ± 5	14%	52%	48% of those with MDD
Direk et al. [44] (2013, Netherlands)	Population	980	72 ± 7	59%	4 ± 2	–	7%, CES-D ≥ 16	None
Moon et al. [30] (2011, South Korea)	Population	123	76 ± 7	26%	3 ± 3	–	47%	None
Pomara et al. [53] (2022, USA)	Population	93	68 ± 6	52%	17 ± 3	24%	52%	None
Sun et al. [38] (2009, USA)	Population	1060	75 ± 9	76%	–	24%	34%	28% of those with MCI

*GDS* Geriatric Depression Scale, *BDI* Beck Depression Inventory, *CES-D* Center for Epidemiologic Studies Depression Scale, *MDD* major depressive disorder, *MCI* mild cognitive impairment, *SCD* subjective cognitive decline.

**Table 2:** Extracted data used in the meta-analyses from the included studies.

Study (Year)	*N*	Amyloid-beta scale	Measurement method	Clinical diagnosis/ depressive symptoms (criteria)	Covariate-adjustment	Cohen’s d ± SE
CSF studies						
Diniz et al. [43] (2014)	50	Continuous	INNO-BIA	Clinical diagnosis (DSM-IV)	No	0.03 ± 0.28 (Aβ42)
Gudmundsson et al. [45] (2007)	84	Continuous	ELISA, Innotest	Clinical diagnosis (DSM-III)	Age	−0.75 ± 0.33 (Aβ42)
				Depressive symptoms (MADRS)		−0.39 ± 0.22 (Aβ42)
Hertze et al. [46] (2010)	66	Continuous	xMAP	Clinical diagnosis (DSM-IV)	No	0.38 ± 0.25 (Aβ42)
						1.10 ± 0.27 (Aβ40)
Hu et al. [51] (2022)	1005	Categorical	ELISA, Innotest	Depressive symptoms (HAMD)	Age, sex, APOE e4	1.06 ± 1.34 (Aβ42)
Krell-Roesch et al. [31] (2022)	784	Continuous	Elecsys	Depressive symptoms (BDI-II)	Age, sex, education, APOE e4	0.37 ± 0.08 (Aβ42)
Marquié et al. [52] (2023)	500	Continuous	ELISA, Innotest	Depressive symptoms (NPI-Q)	No	0.15 ± 0.09 (Aβ42)
Pomara et al. [35] (2012)	47	Continuous	Meso Scale Discovery	Clinical diagnosis (DSM-IV)	Age	0.75 ± 0.31 (Aβ42)
						0.56 ± 0.30 (Aβ40)
Reis et al. [36] (2012)	28	Continuous	ELISA, Innotest	Clinical diagnosis (DSM-IV)	No	0.15 ± 0.42 (Aβ42)
Siafarikas et al. [37] (2021)	142	Continuous	Meso Scale Discovery	Clinical diagnosis (ICD-10)	No	−0.20 ± 0.20 (Aβ42/Aβ40)
PET studies						
Babulal et al. [41] (2020)	301	Categorical	^18^F-Florbetapir	Clinical diagnosis (NACC Form D1)	Age, gender, race, education, APOE e4	−0.15 ± 0.24 (SUVR)
Byun et al. [42] (2016)	56	Continuous	^11^C-PiB	Clinical diagnosis (DSM-IV)	No	0.44 ± 0.27 (SUVR)
Donovan et al. [47] (2015)	248	Continuous	^11^C-PiB	Clinical diagnosis (medical record)	No	0.55 ± 0.26 (DVR)
Feng et al. [48] (2023)	133	Continuous	^11^C-PiB	Clinical diagnosis (DSM-V)	No	−0.26 ± 0.18 (SUVR)
				Depressive symptoms (MADRS)		−0.04 ± 0.18 (SUVR)
Kumar et al. [32] (2011)	39	Continuous	^18^F-FDDNP	Clinical diagnosis (DSM-IV)	No	0.87 ± 0.34 (DVR)
Lewis et al. [33] (2022)	4492	Continuous	^18^F-Florbetapir	Depressive symptoms (GDS)	Race, ethnicity, gender, age, employment, housing situation, marital status, education, alcohol use, smoking, medical morbidity score, exercise per week, sleep per night, history of neurological disease	0.04 ± 0.08 (SUVR)
Moriguchi et al. [34] (2021)	40	Continuous	^11^C-PiB	Clinical diagnosis (DSM-IV)	Age	−0.20 ± 0.32 (SUVR)
Touron et al. [49] (2022)	135	Continuous	^18^F-Florbetapir	Depressive symptoms (GDS)	No	0.10 ± 0.17 (SUVR)
Wang et al. [39] (2021)	235	Continuous	^18^F-Flutemetamol	Depressive symptoms (HAMD)	Age, handedness, education	−0.11 ± 0.13 (SUVR)
Weigand et al. [50] (2022)	703	Categorical	^18^F-Florbetapir/Florbetaben	Depressive symptoms (GDS)	No	0.15 ± 0.08 (SUVR)
Wu et al. [40] (2014)	36	Continuous	^18^F-Florbetapir	Clinical diagnosis (DSM-IV)	No	0.48 ± 0.37 (SUVR)
Plasma studies						
Byun et al. [42] (2016)	56	Continuous	INNO-BIA	Clinical diagnosis (DSM-IV)	No	−0.41 ± 0.27 (Aβ42)
						−0.48 ± 0.27 (Aβ40)
						−0.01 ± 0.27 (Aβ40/Aβ42)
Direk et al. [44] (2013)	980	Continuous	ELISA, EUROIMMUN	Depressive symptoms (CES-D)	Age, gender, education, MMSE score, plasma creatinine levels, antidepressant use	−0.06 ± 0.07 (Aβ42)
						−0.16 ± 0.09 (Aβ40)
Moon et al. [30] (2011)	123	Continuous	ELISA, Biosource	Depressive symptoms (GDS)	No	−0.37 ± 0.18 (Aβ42)
					Age, sex, education	−0.38 ± 0.18 (Aβ42)
Pomara et al. [53] (2022)	93	Continuous	INNO-BIA	Clinical diagnosis (DSM-IV)	Age	−0.52 ± 0.21 (Aβ40)
						0.00 ± 0.21 (Aβ42)
						0.06 ± 0.21 (Aβ42/Aβ40)
Sun et al. [38] (2009)	1060	Continuous	ELISA	Depressive symptoms (CES-D)	Age, race, gender, education, creatinine, cardiovascular disease, APOE e4	0.25 ± 0.07 (Aβ42)
						−0.13 ± 0.21 (Aβ40)

*PET* positron emission topography, *CSF* cerebrospinal fluid, *SE* standard error, *PiB* Pittsburgh compound B, *ELISA* enzyme-linked immunosorbent assay, *DSM* Diagnostic and Statistical Manual of Mental Disorders, *MADRS* Montgomery-Asberg Depression Rating Scale, *GDS* Geriatric Depression Scale, *HAMD* Hamilton Rating Scale for Depression, *BDI* Beck Depression Inventory, *NPI-Q* Neuropsychiatric Inventory Questionnaire, *ICD* International Classification of Diseases, *APOE* apolipoprotein E, *Aβ* amyloid-beta.

The adjusted Newcastle-Ottawa Quality Assessment Scale for cohort studies was used to evaluate the risk of bias, and the included studies scored between four and nine stars on the assessment (Table 3). Regarding selection criteria, nine (38%) studies lost stars as their sample was not representative of community-dwelling older adults without cognitive impairment. Eleven (46%) studies did not adjust for any covariates. All studies ascertained Aβ burden and depression continuously or categorically based on validated cut-off values; the same method to ascertain Aβ burden and depression or depressive symptoms was implemented for depressed cases and healthy controls in each study. Thus, risk of bias based on the ascertainment of outcome was assumed low. Four studies (17%) scored all nine stars. The Egger’s t statistic for the CSF studies (bias = −0.56, SE = 0.97, t(7), = −0.58, *p* = 0.58), PET studies (bias = 0.71, SE = 0.87, t(9) = 0.82, *p* = 0.43), and plasma studies (bias = −1.47, SE = 2.01, t(3), *p* = 0.52) suggested that significant publication bias was unlikely [29].

**Table 3:** Risk of bias assessment using the adjusted Newcastle-Ottawa Quality Assessment Scale Cohort Studies.

Study	Selection			Comparability				Outcome		Total score (max. 9)
	Representative	Selection	Exposure	Age	Sex/gender	Education	Other factors	Outcome	Same method	
Babulal et al. [41]	*	*	*	*	*	*	*	*	*	9
Byun et al. [42]	*	*	*	–	–	–	–	*	*	5
Diniz et al. [43]	*	*	*	–	–	–	–	*	*	5
Direk et al. [44]	*	*	*	*	*	*	*	*	*	9
Donovan et al. [47]	*	*	*	–	–	–	–	*	*	5
Feng et al. [48]	*	*	*	–	–	–	–	*	*	5
Gudmundsson et al. [45]	*	*	*	*	*	–	–	*	*	7
Hertze et al. [46]	*	*	*	–	–	–	–	*	*	5
Hu et al. [51]	–	*	*	*	*	–	*	*	*	7
Krell-Roesch et al. [31]	*	*	*	*	*	*	*	*	*	9
Kumar et al. [32]	–	*	*	*	*	*	–	*	*	7
Lewis et al. [33]	–	*	*	*	*	*	*	*	*	8
Marquié et al. [52]	–	*	*	–	–	–	–	*	*	4
Moon et al. [30]	*	*	*	*	*	*	–	*	*	8
Moriguchi et al. [34]	*	*	*	*	–	–	–	*	*	6
Pomara et al. [35]	–	*	*	*	–	–	–	*	*	4
Pomara et al. [53]	–	*	*	*	–	–	–	*	*	5
Reis et al. [36]	–	*	*	–	–	–	–	*	*	4
Siafarikas et al. [37]	*	*	*	–	–	–	–	*	*	5
Sun et al. [38]	–	*	*	*	*	*	*	*	*	8
Touron et al. [49]	*	*	*	–	–	–	–	*	*	5
Wang et al. [39]	*	*	*	*	*	*	*	*	*	9
Weigand et al. [50]	*	*	*	–	–	–	–	*	*	5
Wu et al. [40]	–	*	*	–	–	–	–	*	*	4

**Fig. 2:**
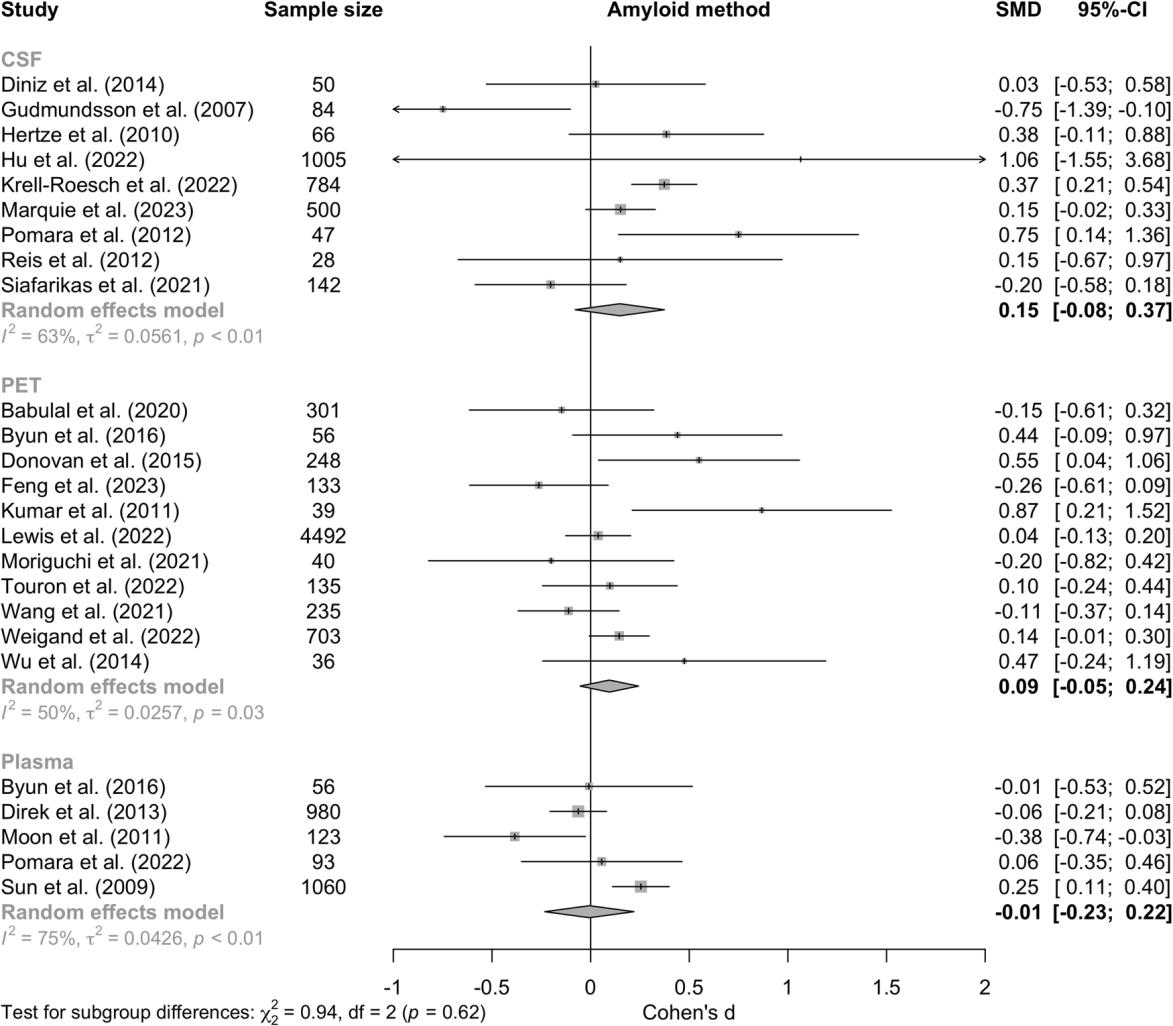
Meta-analyses on the association between amyloid-beta and depression or depressive symptoms using PET, CSF, and plasma. Note: The effect sizes of the individual studies are represented by the squares, of which the size is proportional to the weight of the study. The diamond represents the pooled estimate. The horizontal lines represent the 95% confidence intervals of the individual effect sizes. A positive Cohen’s d represents an association between higher Aβ burden and depression or depressive symptoms. The effect sizes of studies assessing Aβ via cerebrospinal fluid or plasma were flipped.

The characteristics and effect sizes (Cohen’s d ± standard error) of each included study are shown in Table 3. The meta-analysis of the nine CSF studies resulted in an effect size of 0.15 (95% CI: −0.08; 0.37, *p* = 0.20) (Fig. 2). For the 11 PET studies, there was also no association between Aβ burden and depression or depressive symptoms (0.09, 95% CI: −0.05; 0.24, *p* = 0.21). Lastly, for the five plasma studies, no association was found between Aβ burden and depression or depressive symptoms (−0.01, 95% CI: −0.23; 0.22, *p* = 0.96). There was no statistically significant difference between the effect sizes based on how Aβ was assessed (Q(2) = 0.94, *p* = 0.62). However, there was substantial heterogeneity in the CSF (I^2^ = 63%), PET (I^2^ = 50%), and plasma subgroups (I^2^ = 75%).

### Meta-analysis on CSF studies

There were no significant differences between the CSF studies based on covariate adjustment, clinical depression diagnosis versus depressive symptom questionnaire, population-based versus clinical settings, or the inclusion of cognitively impaired individuals vs no cognitive impairment. When removing the one study that only assessed women, a significant association between Aβ burden in CSF and depression or depressive symptoms was found (0.22; 95% CI: 0.04; 0.41, *p* = 0.02). Further, the heterogeneity lessened (I^2^ = 45%) (Supplementary Fig. 1). Results did not change when removing the one study that assessed Aβ categorically. The meta-regression on prevalence of APOE e4 allele or prevalence of individuals with cognitive impairment did not show an effect on the meta-analysis of CSF studies. However, meta-regression reveled that sex/gender did influence the effect size (QM(1) = 6.33, *p* = 0.01) (Supplementary Fig. 2). Further, the R^2^ was 63%, meaning that 63% of the heterogeneity of the meta-analysis on CSF studies could be explained by differences in the sex/gender distribution of the participants. The expected effect size for men was 0.99 (95% CI: 0.33; 1.65), whereas for women it was −1.47 (95% CI: −2.61; −0.32).

### Meta-analysis on PET studies

There was a marginally significant subgroup difference between the PET studies that controlled for covariates and the ones that did not (4 vs. 7 study groups, Q(1) = 3.72, *p* = 0.05). In the studies that did not adjust for covariates, an association was found between Aβ and depression or depressive symptoms (0.24; 95% CI: 0.00; 0.47, *p* < 0.05). Whereas in the covariate-adjusted studies, a null association was found between Aβ and depression or depressive symptoms (Supplementary Fig. 3). When assessing differences between clinical diagnosis or depressive symptom questionnaire, no subgroup differences were found in the PET studies. There was a statistically significant subgroup difference between the PET studies in a clinical setting and those from the general population (6 vs. 5 study groups, Q(1) = 5.67, *p* = 0.02). In the studies from the general population, an association was found between Aβ and depression or depressive symptoms (0.30, 95% CI: 0.06; 0.54, *p* = 0.01). Whereas in the studies from a clinical population, no association was found (Supplementary Fig. 4). There was no subgroup difference between the studies that assessed Aβ categorically or continuously or in the studies that included cognitively impaired individuals and those that did not. The meta-regression did not reveal that sex/gender distribution, prevalence of APOE e4 allele genotype, or prevalence of cognitively impaired individuals influenced the meta-analysis results for the PET studies.

### Meta-analysis on plasma studies

No significant subgroup differences were found between the plasma studies that assessed depression by clinical diagnosis or symptom questionnaire. No studies assessed Aβ categorically; therefore, no subgroup analysis could be done based on Aβ quantification. When removing the one study that was performed in a clinical setting, results remained similar. There was a statistically significant subgroup difference between studies that included cognitively impaired individuals and those that did not (2 vs. 3 study groups, Q(1) = 7.69, *p* < 0.01). There was a statistically significant association between Aβ and depression or depressive symptoms in the studies that included individuals with cognitive impairment (0.24, 95% CI: 0.10; 0.37, *p* = 0.001). In the studies on only cognitively unimpaired individuals, there was no association between Aβ and depression or depressive symptoms (Supplemental Fig. 5). The meta-regression did not reveal that prevalence of APOE e4 allele genotype or inclusion of cognitively impaired individuals influenced the meta-analysis results for the plasma studies. However, meta-regression revealed that sex/gender influenced the effect size of the plasma studies (QM(1) = 14.41, *p* < 0.001) and accounted for the majority of the heterogeneity (Supplemental Fig. 6). However, the opposite was found in plasma compared to in CSF; where the expected effect size for men was −0.80 (95% CI: −1.26; −0.35) and the expected effect size for women was 1.36 (95% CI: 0.66; 2.05).

## Discussion

This systematic review and meta-analysis aimed to explore if depression or depressive symptoms are associated with Aβ burden assessed via CSF, PET, or plasma in older adults without dementia. No association was found between Aβ and depression or depressive symptoms in the CSF, PET, or plasma studies. The Egger’s t-test suggested there was no publication bias. However, there was substantial heterogeneity in the CSF, PET, and plasma studies [29]. Meta-regression revealed that sex/gender distribution in the included studies influenced the effect size in both the CSF and plasma studies and contributed to the heterogeneity. For the PET studies, subgroup analyses revealed that differences in covariate adjustment and in the cohort settings contributed to the heterogeneity. Lastly, subgroup analyses in the plasma studies suggested that differences in studies on cognitively impaired or unimpaired individuals contributed to the heterogeneity.

Two previous systematic reviews have been conducted on Aβ and depression [19, 54], with one including a meta-analysis on CSF and plasma studies [19]. While Nascimento, Silva [19] also did not find an association between CSF levels of Aβ and depression, there was an association between plasma levels of Aβ and depression. However, the included studies in the meta-analysis of Nascimento, Silva [19] included studies assessing serum levels of Aβ, rather than plasma levels. Plasma Aβ levels have been found to be more stable under storage conditions than in serum [55], which was also one of the reasons the current study focused on only plasma assessment of Aβ. The only study that was included both in the current meta-analysis and in the meta-analysis of Nascimento, Silva [19] is Sun, Chiu [38] which was the only included plasma study that found a significant association between Aβ and depressive symptoms. To note, this study was the oldest study of the included plasma studies, and as plasma assays have improved exponentially in the last years for Aβ assessment, this could have explained the discrepancy seen in the previous meta-analysis with the current study.

While this systematic review and meta-analysis did not reveal an overall relationship between Aβ and depression, subgroup analyses revealed an association between depressive symptoms and plasma amyloid-beta in individuals with cognitive impairment. Although not significant, a trend of an association was also found in the CSF and PET studies in those with cognitive impairment. This suggests the possibility that depression may be part of the prodromal stage of dementia, where both pathophysiology (e.g., amyloid burden) and cognitive symptoms are present. Previous studies have also suggested depression as being part of the prodromal stage of dementia [11, 56–58]. In the studies that stratified by cognitive impairment, stronger associations between amyloid burden and depression were also seen in the MCI group compared to those who were cognitively normal. It is possible that amyloid pathology is driving both cognitive and psychological impairment. Future studies should include both repeated measures of Aβ and depressive symptoms to assess their temporal relationship during the extended phases of both preclinical and prodromal stages of dementia.

Due to the low number of studies assessing a longitudinal relationship between Aβ and depression and depressive symptoms, these studies were not included in the systematic review and meta-analysis. While there was a trend towards higher levels of Aβ deposition on PET and depression and depressive symptoms, the current meta-analysis did not find a significant relationship. One longitudinal study did find an association between an increase in depressive symptoms and a higher rate of increase in Aβ deposition on PET [59]. A similar pattern was seen in one longitudinal study on plasma Aβ40/42, where no baseline association was found with depressive symptoms, but a longitudinal association was found with plasma Aβ40/42 and depressive symptoms nine years later [60]. Another recent study also found an association with plasma Aβ42/40 and depressive symptoms longitudinally, both in one year and in three years [53].

Further, some articles could not be included due to insufficient information to calculate an effect size. These studies also did not find an association between Aβ and depression or depressive symptoms [47, 61–65]. However, they also focused on individuals without objective cognitive impairment. However, two studies that looked regionally found higher levels of amyloid deposition based on PET imaging in either the temporal, parietal, and occipital areas in those who have a late-life depression diagnosis compared to non-depressed controls [66] or in just the medial temporal region in those with depressive symptoms [67]. The current meta-analysis focused only on total rather than regional levels of amyloid in PET. It is possible that depression or depressive symptoms is associated first with amyloid deposition in temporal regions, which is why our current meta-analysis on PET studies found a null result. Future studies should elucidate this possible region-specific association between depression or depressive symptoms and amyloid accumulation.

This systematic review and meta-analysis had some limitations. The current study focused on cross-sectional studies; therefore, the temporal relationship between amyloid burden and depression or depressive symptoms could not be elucidated. Of note, only four studies reported the ethnicity of study participants, and participants were mostly Caucasian. This is of importance as the limited ethnicities could restrict the generalizability of our findings. However, this study also had many strengths. We assessed multiple methods to assess Aβ burden, used a random-effects meta-analysis, and performed multiple subgroup analyses to elucidate the heterogeneity in the meta-analyses.

In conclusion, this meta-analysis demonstrated no evidence of an association between depression or depressive symptoms and Aβ in CSF, PET, or plasma in older adults without dementia. However, our subgroup analyses suggested a relationship during the stage of cognitive impairment. It is possible that late-life depressive symptoms are driven by amyloid accumulation during the prodromal stage of dementia, when cognitive impairment becomes apparent. More longitudinal studies with repeated measurements are needed to discover if depression is a reaction to the development of cognitive decline symptoms in late-life or independently driven by amyloid accumulation.

### Supplementary information

Supplemental Material

## Data Availability

The data that support the findings of this study are available from the corresponding author upon reasonable request.

## Code availability

The code is available by request to the corresponding author.
