# Supplementary material for: Association of amyloid-beta with depression or depressive symptoms in older adults without dementia: a systematic review and meta-analysis
Source: Transl Psychiatry. 2024 Jan 15;14:25. doi: 10.1038/s41398-024-02739-9 (PMC10789765; doi:10.1038/s41398-024-02739-9)

**Supplemental Info**

**Supplementary Info 1**

("Depression"[MeSH Terms] OR "Depressive Disorder"[MeSH Terms] OR "depress*"[Title/Abstract] OR "Depression"[Title/Abstract] OR "Depressions"[Title/Abstract] OR "Depressive"[Title/Abstract] OR "Depressed"[Title/Abstract] OR "affective disorder"[Title/Abstract] OR "Dysphoria"[Title/Abstract] OR "Dysthymia"[Title/Abstract] OR "depressed mood"[Title/Abstract] OR "mood disorder"[Title/Abstract] OR "depressive symptoms"[Title/Abstract]) AND (("Amyloid"[MeSH Terms] OR "plaque, amyloid"[MeSH Terms] OR "amyloid*"[Title/Abstract] OR "AB"[Title/Abstract] OR "Abeta40"[Title/Abstract] OR "Abeta42"[Title/Abstract] OR "abeta 40"[Title/Abstract] OR "abeta 42"[Title/Abstract] OR "betaA"[Title/Abstract] OR "betaA40"[Title/Abstract] OR "betaA42"[Title/Abstract] OR "betaa 40"[Title/Abstract] OR "betaa 42"[Title/Abstract] OR "beta amyloid"[Title/Abstract] OR "Abeta"[Title/Abstract] OR "Abeta40"[Title/Abstract] OR "Abeta42"[Title/Abstract] OR "abeta 40"[Title/Abstract] OR "abeta 42"[Title/Abstract] OR "PIB"[Title/Abstract] OR "pittsburgh compound b"[Title/Abstract] OR "flutemetamol"[Title/Abstract] OR "florbetapir"[Title/Abstract] OR "florbetaben"[Title/Abstract] OR "senile plaque*"[Title/Abstract]) AND ("Positron-Emission Tomography"[MeSH Terms] OR "PET"[Title/Abstract] OR "positron emission tomograph*"[Title/Abstract] OR "Cerebrospinal Fluid"[MeSH Terms] OR "CSF"[Title/Abstract] OR "cerebrospinal fluid*"[Title/Abstract] OR "cerebro spinal fluid*"[Title/Abstract] OR "plasma"[Title/Abstract] OR "plasmas"[Title/Abstract] OR "neuropatholog*"[Title/Abstract] OR "amyloid patholog*"[Title/Abstract] OR "Neuropathology"[MeSH Terms]))

**Supplementary Info 2**

**Adjusted version of the Newcastle-Ottawa Quality Assessment Scale for Cohort Studies**

Note: A study can be awarded a maximum of one star for each numbered item within the Selection and Outcome categories. A maximum of two stars can be given for Comparability. Total maximum number of stars is nine.

**Selection criteria**

1) Representativeness of the exposed cohort (depressed, without dementia)

a) truly representative of the average older adult without dementia in the community (i.e., community-based cohort, can include individuals with MCI or subjective cognitive decline as well) *

b) somewhat representative of the average older adult without dementia in the community (e.g., inclusion criteria regarding only individuals without MCI) *

c) selected group of users, e.g., volunteers, memory clinic visitors, only individuals at higher risk (e.g., only subjective complaints, only APOE e4 carriers)

d) no description of the derivation of the cohort

2) Selection of the non-exposed cohort (non-depressed, without dementia)

a) drawn from the same community as the exposed cohort *

b) drawn from a different source

c) no description of the derivation of the non-exposed cohort

3) Ascertainment of exposure

a) clinical interview *

b) established depressive symptom questionnaire *

c) categorized based on established or published cut-offs for a symptom questionnaire *

d) categorized based on non-established cut-offs (e.g., z-score cut-off, mean split, median split)

e) no description

**Comparability**

1) Comparability of cohorts regarding the design or analysis

a) study controls for age *

b) study controls for sex/gender *

c) study controls for education *

d) study controls for any additional factor *

**Outcome (Amyloid)**

1) Ascertainment of outcome

a) via PET scan *

b) via CSF *

c) via plasma *

d) no description

2) Same method of assessment for cases (depressed) and controls (non-depressed)

a) yes *

b) no

**Supplementary Figure 1.** Sensitivity analysis on CSF studies when removing one cohort that consisted only of women (*Gudmundsson et al*.).


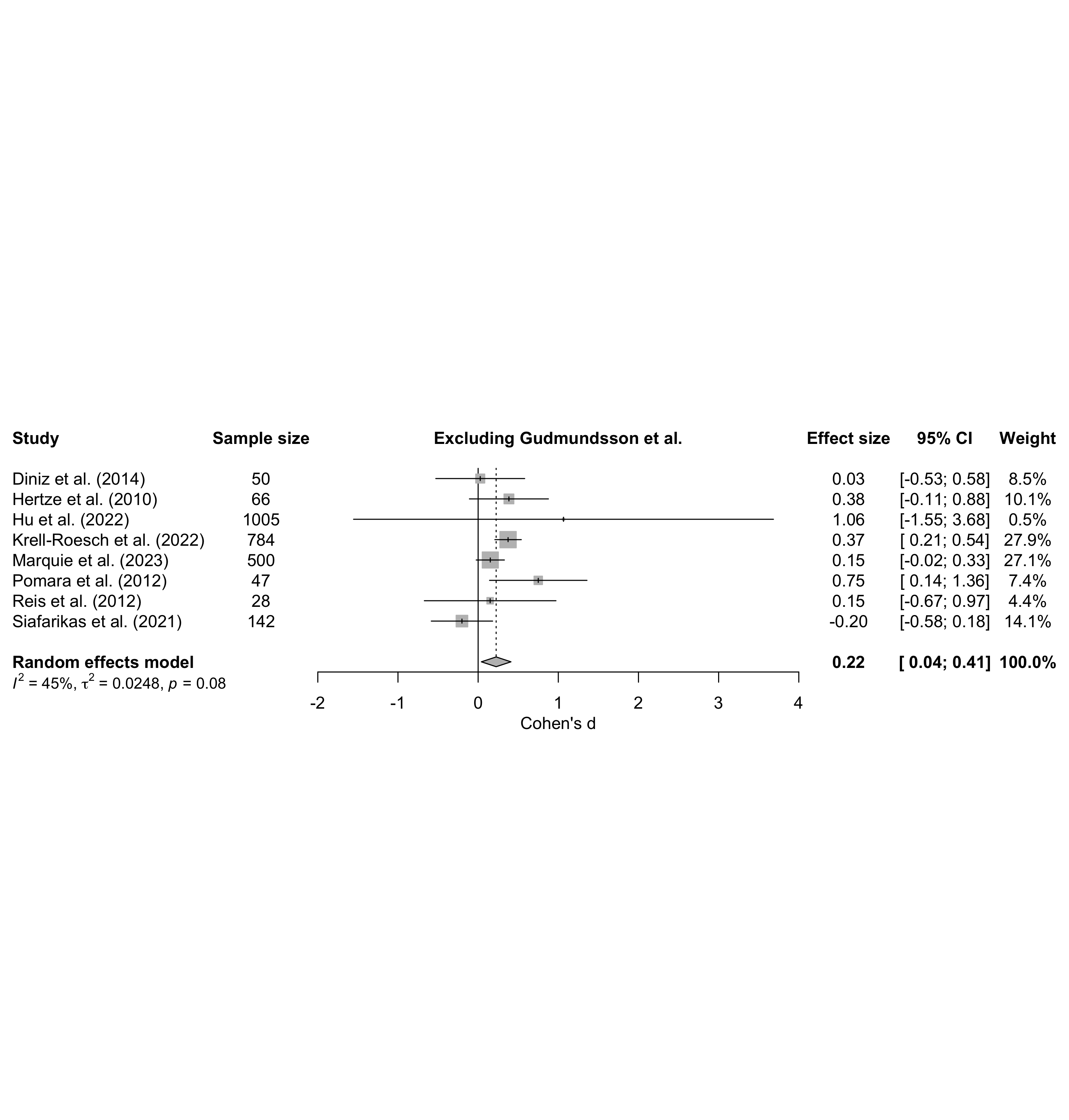


**Supplementary Figure 2.** Bubble plot representation on the meta-regression of proportion of women in the CSF studies with Cohen’s d.


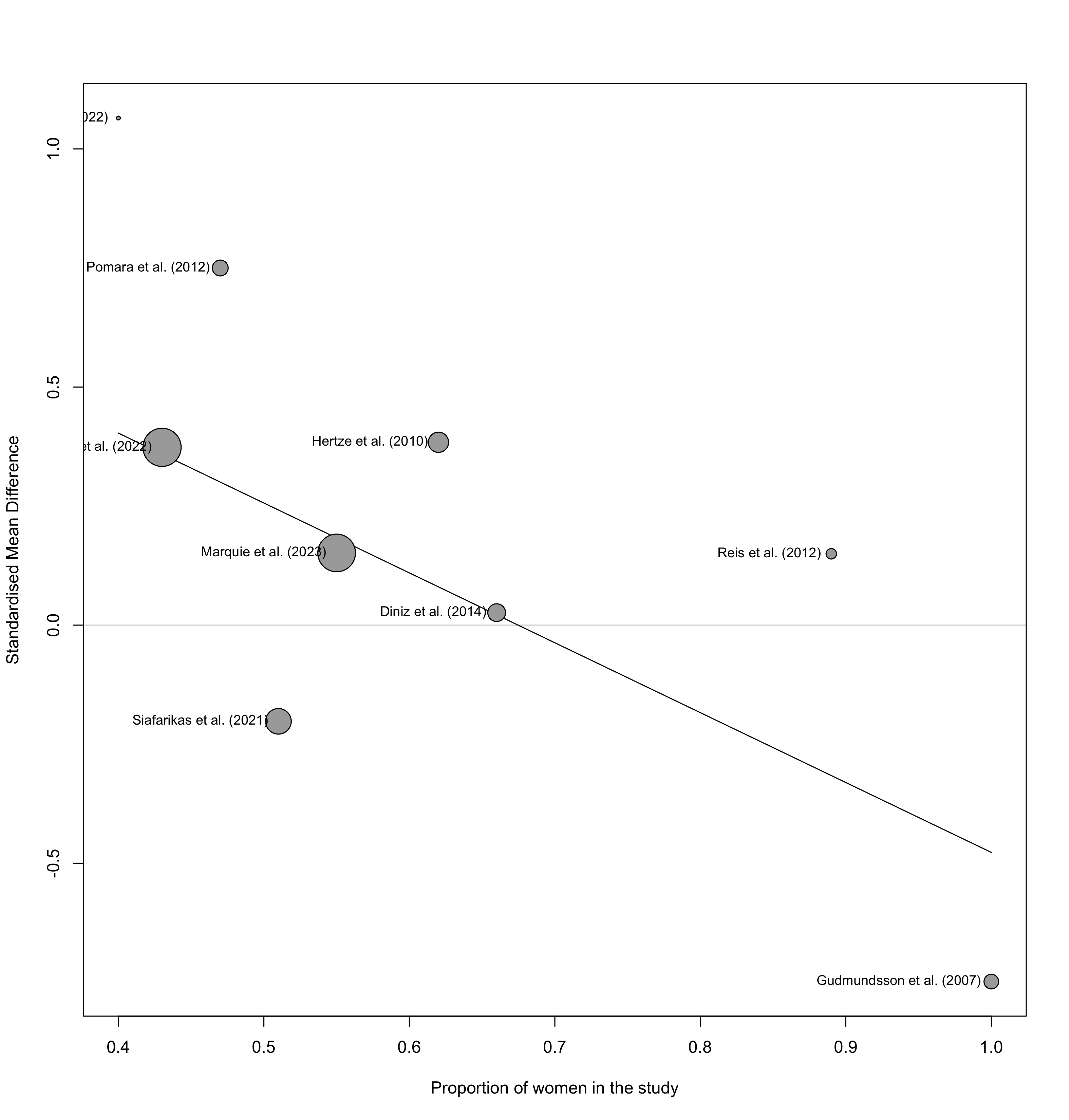


**Supplementary Figure 3.** Subgroup analysis on the PET studies that adjusted for covariates and the studies that did not adjust for covariates.


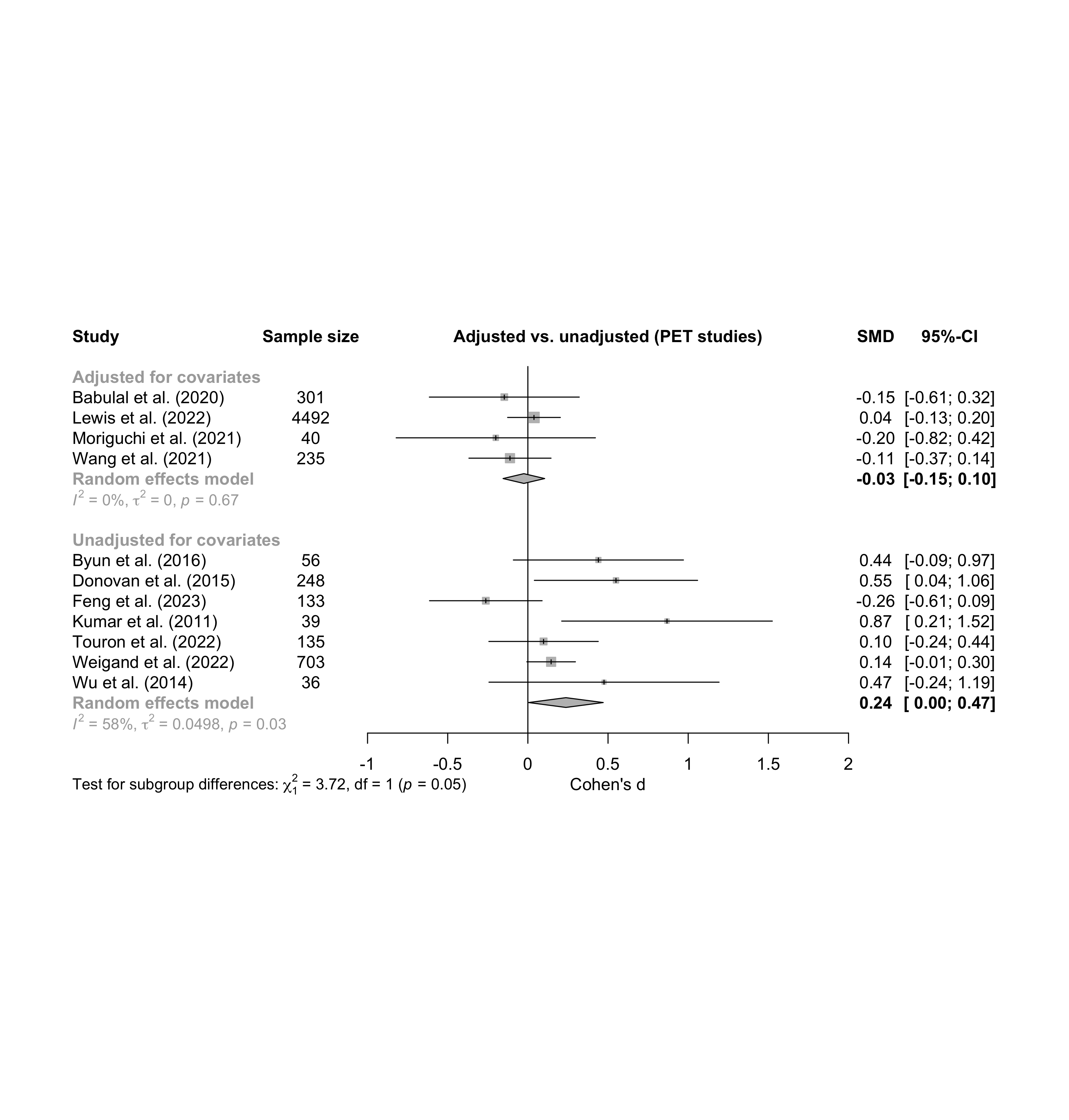


**Supplementary Figure 4.** Subgroup analysis on the PET studies that included cohorts from the general population versus those within a clinical setting.


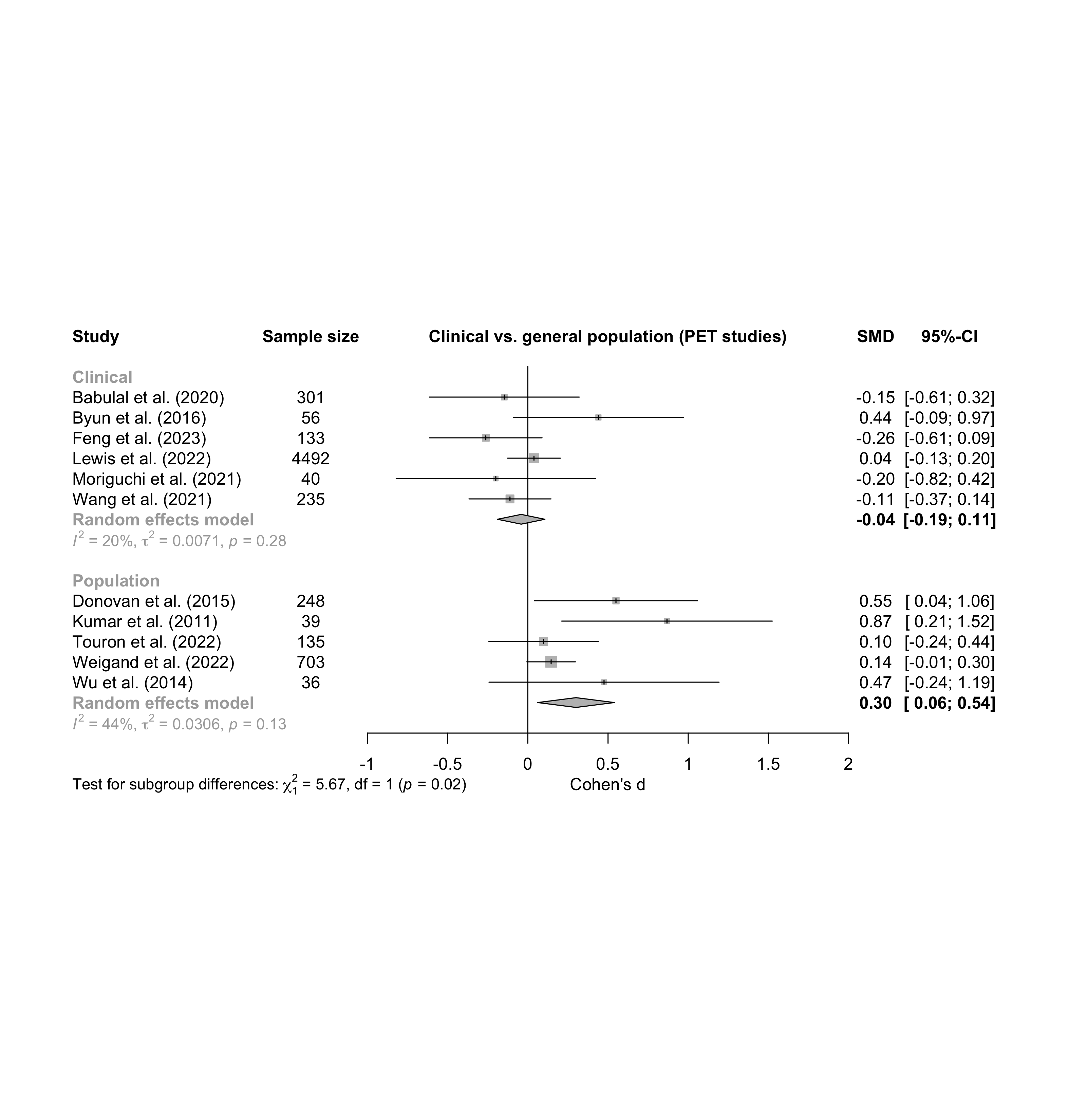


**Supplementary Figure 5.** Subgroup analysis on the plasma studies that included cognitively unimpaired individuals and the studies only on cognitively unimpaired individuals.


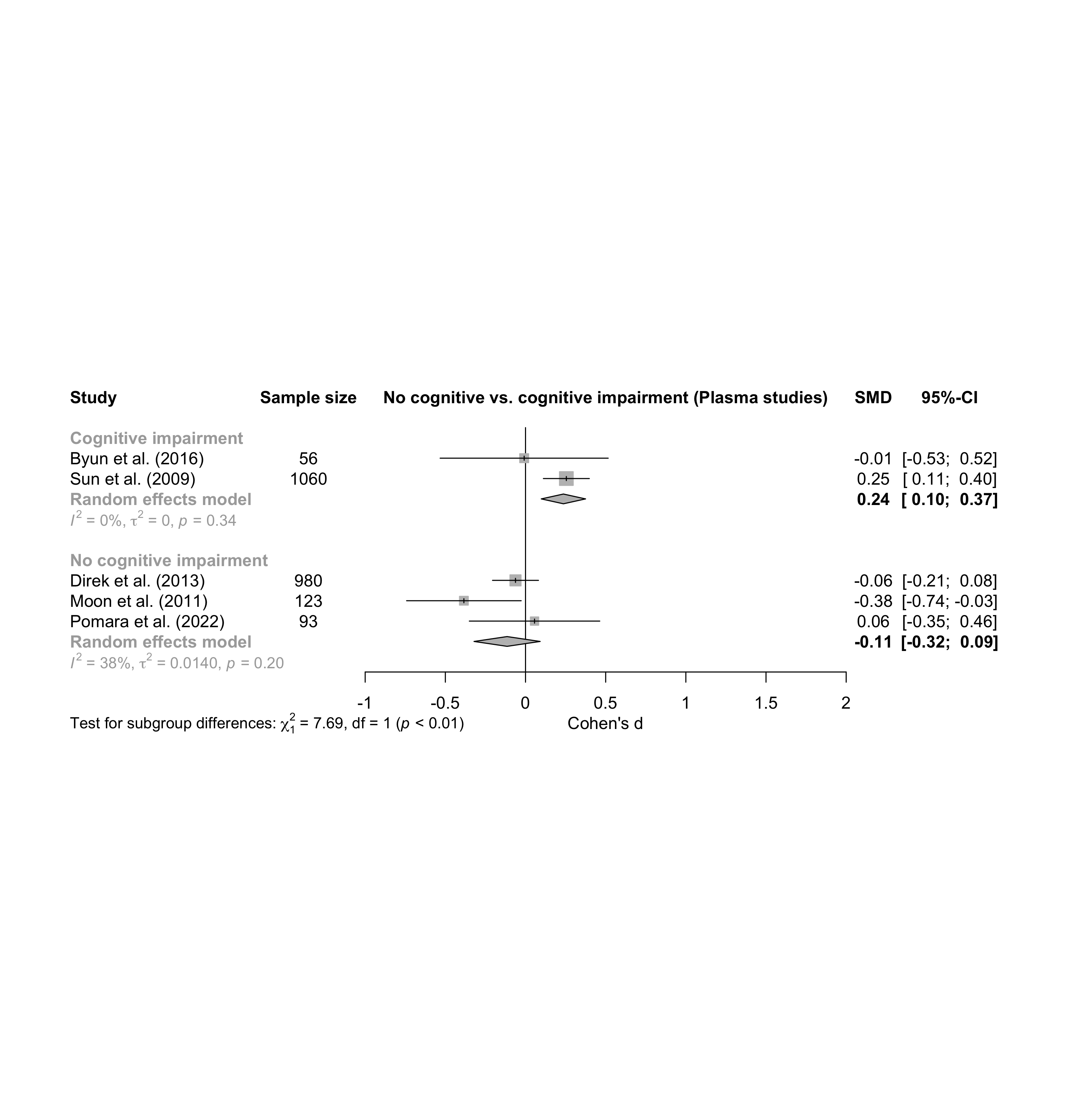


**Supplementary Figure 6.** Bubble plot representation on the meta-regression of proportion of women in the plasma studies with Cohen’s d.


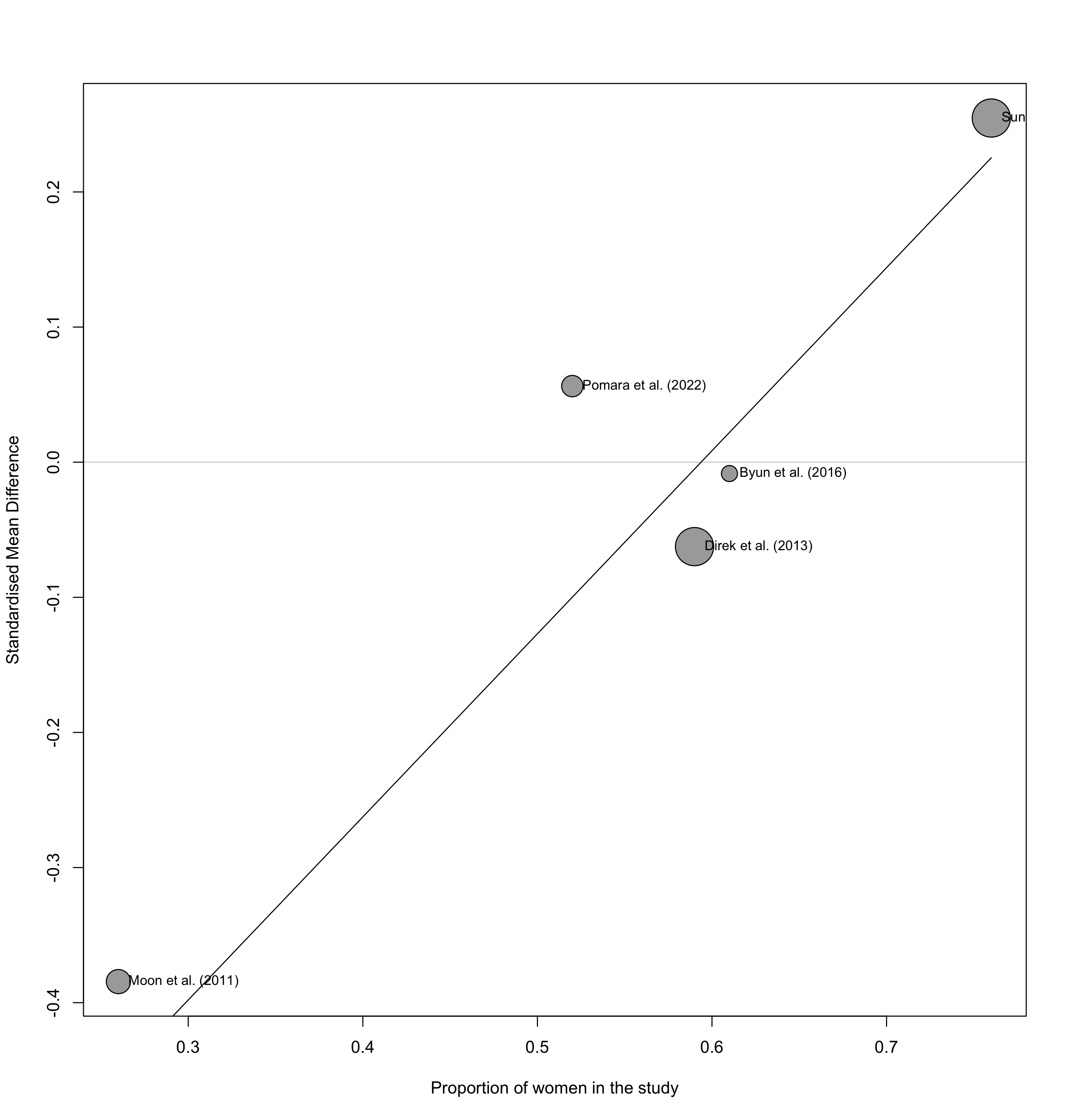

Supplement: Supplementary file 1 — Supplemental Material [file 41398_2024_2739_MOESM1_ESM.docx]
